# A new species of the genus *Kurixalus* from Yunnan, China (Anura, Rhacophoridae)

**DOI:** 10.3897/zookeys.694.12785

**Published:** 2017-08-29

**Authors:** Guohua Yu, Jishan Wang, Mian Hou, Dingqi Rao, Junxing Yang

**Affiliations:** 1 Kunming Institute of Zoology, Chinese Academy of Sciences, 32 Jiaochang Donglu, Kunming, Yunnan 650223, China; 2 Kunming College of Life Science, University of Chinese Academy of Sciences, Kunming, Yunnan 650204, China; 3 Kunming Institute of survey & design, State Forestry Administration, Kunming, Yunnan 650216, China; 4 Institute of Continuing Education, Sichuan Normal University, Chengdu, Sichuan 610068, China

**Keywords:** China, *Kurixalus lenquanensis* sp. n., Yunnan

## Abstract

A new species of the genus *Kurixalus* (Anura: Rhacophoridae) is described from Yunnan, China based on morphological and molecular evidence. The new species, *Kurixalus lenquanensis* **sp. n.**, is distinguished from other congeneric species by a combination of (1) smaller body size (SVL in males ranges from 25.0 to 28.9 mm), (2) obtusely pointed snout with no prominence on tip, (3) rough and brown dorsum with darker mark, (4) absence of large dark spots on ventral surface, (5) brownish clouded chin, (6) granular throat, chest, and belly, (7) presence of vomerine teeth, (8) serrated dermal fringes along outer edge of limbs, (9) slight nuptial pad, (10) golden brown iris, and (11) single internal vocal sac. The new species is known only from montane scrub vegetation at the type locality (Lenquan Village, Mengzi City, Yunnan Province) and Yangjiatian Village, Gejiu City, Yunnan Province. Genetically, the new species is nested within a clade of Taiwanese *Kurixalus* and recovered as the sister taxon to *Kurixalus idiootocus* with strong support values, indicating that the ancestor of this new species might have come from Taiwan Island or the ancestor of this new species may have been widespread in southern China and the descendent species in between Taiwan and Yunnan has become extinct.

## Introduction

The genus *Kurixalus* Ye, Fei, & Dubois in Fei 1999 has a wide distribution including eastern India, Indochina, Sunda islands, Philippine archipelago, montane southern China, and adjacent continental islands (Hainan, Taiwan, and Ryukyu Islands); currently 14 species are recognized in this genus (Frost 2017). Owing to its morphological conservativeness, the taxonomy and systematics of this genus was once very confusing. Inger et al. (1999) considered that Chinese and Vietnamese *Kurixalus odontotarsus* (Ye & Fei in Ye et al. 1993) probably belong to *Kurixalus verrucosus* (Boulenger, 1893) or *Kurixalus bisacculus* (Taylor, 1962), Orlov et al. (2002) also considered Vietnamese *K. odontotarsus* as *K. verrucosus*, and *Kurixalus hainanus* (Zhao, Wang, & Shi in Zhao et al. 2005) was thought to be a synonym of *K. odontotarsus* by some authors (e.g. Fei 1999, Fei et al. 2010). However, based on evidence from molecular data, Yu et al. (2010) proposed that *K. odontotarsus*, *K. bisacculus*, and *K. verrucosus* should be treated as three independent species and suggested placing *K. odontotarsus* from Tibet and *K. hainanus* in *K. verrucosus* and *K. bisacculus*, respectively. Moreover, Yu et al. (2010) considered that the distributional range of *K. bisacculus* should be expanded to include most regions of South China. Thus, currently three species of *Kurixalus* (*K. odontotarsus*, *K. bisacculus*, and *K. verrucosus*) are recognized in mainland China and two of them (*K. odontotarsus* and *K. bisacculus*) exist in Yunnan according to Yu et al. (2010).

**Figure 1.**
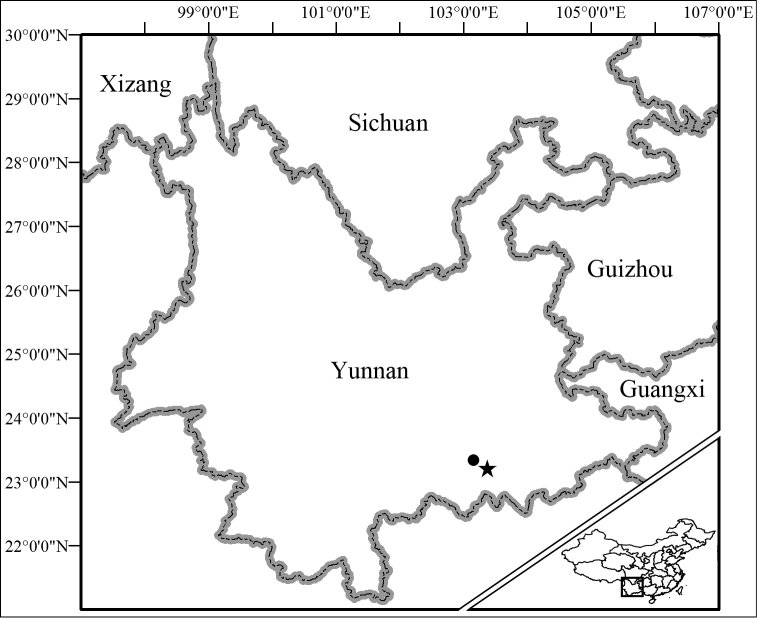
Known distribution of *Kurixalus lenquanensis* sp. n. Filled star indicates the type locality and filled circle indicates Yangjiatian Village.

During recent fieldwork in Yunnan, China (Fig. 1), a small tree frog was discovered that is morphologically similar in appearance with other *Kurixalus* species from Yunnan. It appears to be closely related to *Kurixalus idiootocus* (Kuramoto & Wang, 1987) from Taiwan based on molecular phylogenetic analyses, but it is different from *K. idiootocus* based on the following characters: obtusely pointed snout with no prominence on tip, single internal vocal sac, loreal region oblique, absence of a pair of symmetrical large dark patches on chest, and absence of supernumerary plantar tubercles.

## Materials and methods

**Sampling**. Specimens were collected during fieldwork in Honghe Autonomous Prefecture, Yunnan, China in 2015 and 2016. They were euthanized with diethyl ether anesthesia and fixed by 90% ethanol before being stored in 70% ethanol. Liver tissues were preserved in 99% ethanol. Specimens and tissue samples were deposited at Kunming Institute of Zoology, Chinese Academy of Sciences (KIZ 170175Y–170186Y) and Kunming Institute of Survey & Design, State Forestry Administration (KISD 1506203–1506204).

**Morphology**. The preserved specimens were examined, measured, and compared with available specimens and published descriptions of currently recognized species of *Kurixalus* from China and neighboring countries (Günther 1858, Boulenger 1893, Annandale 1912, Kuramoto and Wang 1987, Inger et al. 1999, Bossuyt and Dubois 2001, Matsui and Orlov 2004, Mathew and Sen 2008, Nguyen et al. 2014a, Nguyen et al. 2014b, Wu et al. 2016). Measurements were taken using digital calipers to the nearest 0.1 mm. Morphological terminology follows Fei (1999). Measurements include: snout-vent length (**SVL**, from tip of snout to vent); head length (**HL**, from tip of snout to rear of jaws); head width (**HW**, width of head at its widest point); snout length (**SL**, from tip of snout to anterior border of eye); internarial distance (**IND**, distance between nares); interorbital distance (**IOD**, minimum distance between upper eyelids); upper eyelid width (**UEW**, maximum width of upper eyelid); eye diameter (**ED**, diameter of exposed portion of eyeball); tympanum diameter (**TD**, the greater of tympanum vertical and horizontal diameters); distance from nostril to eye (**DNE**, from nostril to anterior border of eye); forelimb length (**FLL**, from elbow to tip of third finger); tibia length (**TL**, distance from knee to heel); foot length (**FL**, from proximal end of inner metatarsal tubercle to tip of fourth toe); and thigh length (**THL**, from vent to knee).

A multivariate principal component analysis (PCA) was conducted using SPSS 17.0 (SPSS Inc.) based on a correlation matrix of size-standardized measurements (all measurements divided by SVL). Scatter plots of the scores of the first two factors of PCA were used to examine the differentiation among the new species, *K. idiootocus*, *K. bisacculus*, and *K. odontotarsus*.

**Molecular analyses**. In order to support the generic placement of the new species, the phylogenetic position of the new species was investigated based on molecular data. Total genomic DNA was extracted from liver tissue stored in 99% ethanol. Tissue samples were digested using proteinase K, and subsequently purified following a standard phenol/chloroform isolation and ethanol precipitation. A fragment of the mitochondrial 16S rRNA gene was amplified and sequenced. The primers and experiment protocols used in this study are the same as with Yu et al. (2010). Homologous sequences of other *Kurixalus* species were obtained from GenBank and all new sequences have been deposited in GenBank under Accession Nos. KY768931–KY768944 (Table 1). *Philautus abditus* Inger, Orlov, & Darevsky, 1999 and *Raorchestes menglaensis* (Kou, 1990) were selected as outgrups based on Yu et al. (2013).

**Table 1.** Sequences used in this study.

Species	Locality	Voucher no.	GenBank no.
*Philautus abditus*	Krong Pa, Gia Lai, Vietnam	ROM 33145	GQ285673
*Raorchestes menglaensis*	Lvchuan, Yunnan, China	060821286Rao	GQ285676
*Kurixalus appendiculatus*	Bukit Sarang, Sarawak, Malaysia	FMNH 267896	JQ060937
*Kurixalus eiffingeri*	Okinawa Islands, Japan	KUHE 12910	AB933305
*Kurixalus idiootocus*	Wulai, Taipei, Taiwan	A127	DQ468674
*Kurixalus idiootocus*	Nan-Tou, Tung Fu, Taiwan	UMFS 5702	DQ283054
*Kurixalus idiootocus*	Taiwan	KUHE 12979	AB933306
*Kurixalus idiootocus*	Taiwan	SCUM 061107L	EU215547
*Kurixalus idiootocus*	Taiwan	CAS 211366	AF458129
*Kurixalus berylliniris*	Beinan, Taitung, Taiwan	11311 (CE01X)	DQ468669
*Kurixalus wangi*	Shouka, Pintung, Taiwan	11328 (CE06)	DQ468671
*Kurixalus banaensis*	Krong Pa, Gia Lai, Vietnam	ROM 32986	GQ285667
*Kurixalus viridescens*	Hon Ba, Khanh Hoa, Vietnam	VNMN 03802	AB933284
*Kurixalus motokawai*	Kon Tum, Vietnam	VNMN 03458	LC002888
*Kurixalus verrucosus*	Nagmung, Kachin, Myanmar	CAS 224381	GU227329
*Kurixalus verrucosus*	Nagmung, Kachin, Myanmar	CAS 224563	GU227330
*Kurixalus verrucosus*	Nagmung, Kachin, Myanmar	CAS 225128	GU227331
*Kurixalus verrucosus*	Tai Nai, Kachin, Myanmar	CAS 231489	GU227332
*Kurixalus verrucosus*	Mohynin, Kachin, Myanmar	CAS 231491	GU227333
*Kurixalus odontotarsus*	Mengyang, Yunnan, China	YGH 090175	GU227282
*Kurixalus odontotarsus*	Mengyang, Yunnan, China	YGH 090176	GU227283
*Kurixalus odontotarsus*	Caiyanghe, Yunnan, China	YGH 090131	GU227290
*Kurixalus odontotarsus*	Caiyanghe, Yunnan, China	KIZ 060821030	GU227294
*Kurixalus bisacculus*	Pingbian, Yunnan, China	YGH 080166	GU227295
*Kurixalus bisacculus*	Pingbian, Yunnan, China	YGH 080168	GU227296
*Kurixalus bisacculus*	Jinping, Yunnan, China	KIZ 060821124	KX554511
*Kurixalus bisacculus*	Thanh Hoa, Vietnam	VNMN 03808	AB933294
*Kurixalus bisacculus*	Wenshan, Yunnan, China	KIZ 3315	GU227305
*Kurixalus bisacculus*	Wenshan, Yunnan, China	KIZ 3317	GU227306
*Kurixalus bisacculus*	Wenshan, Yunnan, China	YGH 090044	GU227299
*Kurixalus bisacculus*	Wenshan, Yunnan, China	YGH 090046	GU227300
*Kurixalus bisacculus*	Jingxi, Guangxi, China	YGH 090280	GU227313
*Kurixalus bisacculus*	Jingxi, Guangxi, China	YGH 090281	GU227314
*Kurixalus bisacculus*	Libo, Guizhou, China	YGH 090081	GU227307
*Kurixalus bisacculus*	Nanning, Guangxi, China	YGH 090268	GU227310
*Kurixalus bisacculus*	Nanning, Guangxi, China	YGH 090270	GU227312
*Kurixalus bisacculus*	Jinxiu, Guangxi, China	KIZ 060821015	GU227319
*Kurixalus bisacculus*	Longmeng, Guangdong, China	YGH 090201	GU227320
*Kurixalus bisacculus*	Longmeng, Guangdong, China	YGH 090202	GU227321
*Kurixalus bisacculus*	Mt. Wuzhi, Hainan, China	MVZ Herp 236722	JQ060928
*Kurixalus bisacculus*	Bawangling, Hainan, China	MVZ Herp 236725	JQ060929
*Kurixalus bisacculus*	Ha Giang, Vietnam	VNMN 01561	AB933287
*Kurixalus bisacculus*	Pac Ban, Tuyen Quang, Vietnam	ROM 30042	KC465809
*Kurixalus bisacculus*	Cao Bang, Vietnam	ROM 36726	KC465802
*Kurixalus bisacculus*	Cao Bang, Vietnam	VNMN 03805	AB933288
*Kurixalus bisacculus*	Tam Dao, Vinh Phu, Vietnam	MVZ Herp 223857	JQ060931
*Kurixalus bisacculus*	Tam Dao, Vinh Phu, Vietnam	MVZ Herp 226463	JQ060932
*Kurixalus bisacculus*	Chi Linh, Hai Duong, Vietnam	ROM 36829	KC465812
*Kurixalus bisacculus*	Chi Linh, Hai Duong, Vietnam	ROM 36827	KC465813
*Kurixalus bisacculus*	Pua, Nan, Thailand	THNHM 10051	GU227334
*Kurixalus bisacculus*	Pua, Nan, Thailand	THNHM 10052	GU227335
*Kurixalus baliogaster*	Kon Tum, Vietnam	VNMN 03618	AB933300
*Kurixalus baliogaster*	Gia Lai, Vietnam	VNMN 03812	AB933298
*Kurixalus lenquanensis* sp. n.	Lenquan, Mengzi, Yunnan, China	KIZ 170175Y	KY768931
*Kurixalus lenquanensis* sp. n.	Lenquan, Mengzi, Yunnan, China	KIZ 170176Y	KY768932
*Kurixalus lenquanensis* sp. n.	Lenquan, Mengzi, Yunnan, China	KIZ 170177Y	KY768933
*Kurixalus lenquanensis* sp. n.	Lenquan, Mengzi, Yunnan, China	KIZ 170178Y	KY768934
*Kurixalus lenquanensis* sp. n.	Lenquan, Mengzi, Yunnan, China	KIZ 170179Y	KY768935
*Kurixalus lenquanensis* sp. n.	Lenquan, Mengzi, Yunnan, China	KIZ 170180Y	KY768936
*Kurixalus lenquanensis* sp. n.	Lenquan, Mengzi, Yunnan, China	KIZ 170181Y	KY768937
*Kurixalus lenquanensis* sp. n.	Lenquan, Mengzi, Yunnan, China	KIZ 170182Y	KY768938
*Kurixalus lenquanensis* sp. n.	Lenquan, Mengzi, Yunnan, China	KISD 1506203	KY768939
*Kurixalus lenquanensis* sp. n.	Lenquan, Mengzi, Yunnan, China	KISD 1506204	KY768940
*Kurixalus lenquanensis* sp. n.	Yangjiatian, Gejiu, Yunnan, China	KIZ 170183Y	KY768941
*Kurixalus lenquanensis* sp. n.	Yangjiatian, Gejiu, Yunnan, China	KIZ 170184Y	KY768942
*Kurixalus lenquanensis* sp. n.	Yangjiatian, Gejiu, Yunnan, China	KIZ 170185Y	KY768943
*Kurixalus lenquanensis* sp. n.	Yangjiatian, Gejiu, Yunnan, China	KIZ 170186Y	KY768944

Sequences were aligned using CLUSTAL X v1.83 (Thompson et al. 1997) with the default parameters and the alignment was revised by eye. Pairwise distances between species were calculated in MEGA 5 (Tamura et al. 2011). The best substitution model was selected using the Akaike Information Criterion (AIC) in MODELTEST v3.7 (Posada and Crandall 1998). Bayesian phylogenetic inference and Maximum likelihood analysis were performed in MRBAYES 3.1.2 (Huelsenbeck and Ronquist 2001) and RAXMLGUI 1.5b1 (Silvestro and Michalak 2012), respectively, based on the selected substitution model. For the Bayesian analysis, two runs were performed simultaneously with four Markov chains starting from random tree. The chains were run for 4,000,000 generations and sampled every 100 generations. The first 25% of the sampled trees was discarded as burn-in after the standard deviation of split frequencies of the two runs was less than a value of 0.01, and then the remaining trees were used to create a 50% majority-rule consensus tree and to estimate Bayesian posterior probabilities (BPPs). For the maximum likelihood analysis, node support was estimated by 1,000 rapid bootstrap replicates.

## Results

**Figure 2.**
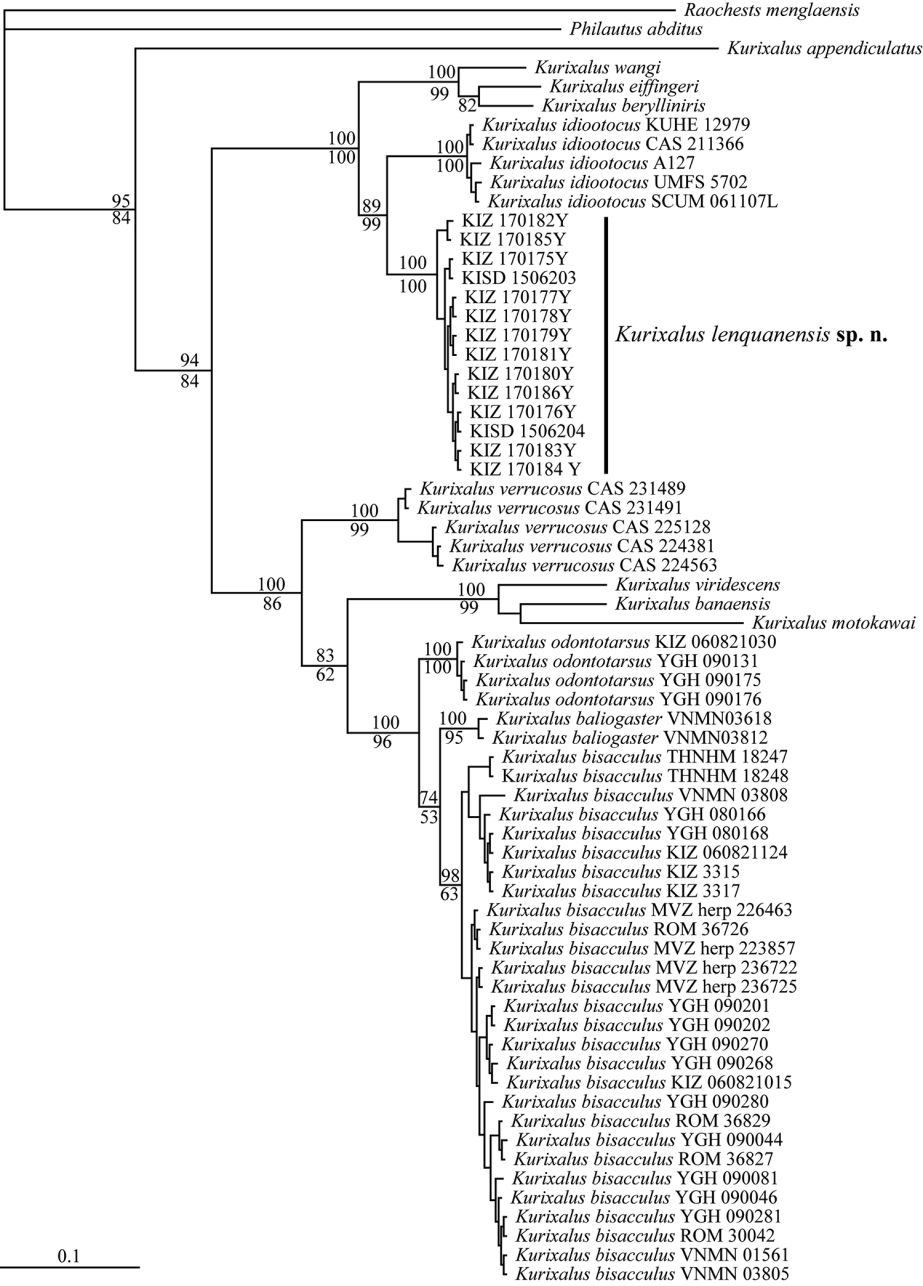
Bayesian phylogram of *Kurixalus* inferred from 870 bp of 16S rRNA gene. Numbers above and below branches are Bayesian posterior probabilities (BPP) and ML bootstrap values (only values above 50% are shown), respectively.

**Molecular data.** The obtained sequence alignment is 870 bp in length. Both Bayesian and maximum likelihood analyses strongly supported that *Kurixalus lenquanensis* sp. n. is in the genus *Kurixalus* and is the sister taxon to *K. idiootocus* (Fig. 2). The average divergence (p-distance) between the new species and other congeneric species ranged from 4.72% to 16.84% (Table 2). This level of divergence in the 16S rRNA gene is indicative of differentiation at the species level in frogs (Fouquet et al. 2007) and is even higher than between other recognized congeners of *Kurixalus* (i.e. *Kurixalus eiffingeri* [Boettger, 1895]/*Kurixalus berylliniris* Wu, Huang, Tsai, Li, Jhang, & Wu, 2016, *K. eiffingeri*/*Kurixalus wangi* Wu, Huang, Tsai, Li, Jhang, & Wu, 2016, *K. berylliniris*/*K. wangi*, *K. bisacculus*/*K. odontotarsus*, *Kurixalus baliogaster* [Inger, Orlov, & Darevsky, 1999]/*K. odontotarsus*, and *K. baliogaster*/*K. bisacculus*; Table 2).

**Table 2.** Average uncorrected p-distances (%) among members of *Kurixalus* calculated from 16S rRNA gene sequences. Average intraspecific differences (p-distance, %) are shown on the diagonal.

**Species**	**1**	**2**	**3**	**4**	**5**	**6**	**7**	**8**	**9**	**10**	**11**	**12**	**13**
1. *K. appendiculatus*	–												
2. *K. eiffingeri*	17.48	–											
3. *K. idiootocus*	17.71	8.07	0.18										
4. *K. berylliniris*	16.29	3.23	6.14	–									
5. *K. wangi*	17.05	3.61	6.52	3.98	–								
6. *K. banaensis*	16.71	12.98	12.48	10.82	11.01	–							
7. *K. viridescens*	18.95	12.76	12.82	11.79	11.41	5.96	–						
8. *K. motokawai*	18.57	14.85	14.06	12.74	12.93	8.76	9.35	–					
9. *K. verrucosus*	16.50	11.70	10.61	10.28	8.96	10.16	10.55	12.87	1.07				
10. *K. odontotarsus*	17.62	12.03	11.60	10.74	10.45	9.02	9.27	11.71	7.22	0.06			
11. *K. bisacculus*	17.59	12.86	12.35	11.65	11.08	9.85	9.77	11.90	7.87	3.03	0.86		
12. *K. baliogaster*	17.55	12.20	11.92	11.59	10.64	9.52	9.18	11.85	8.20	3.44	2.98	0.35	
13. *K. lenquanensis* sp. n.	16.84	7.49	4.72	5.56	5.94	12.51	12.94	14.38	10.47	11.02	11.47	10.98	0.23

**Figure 3.**
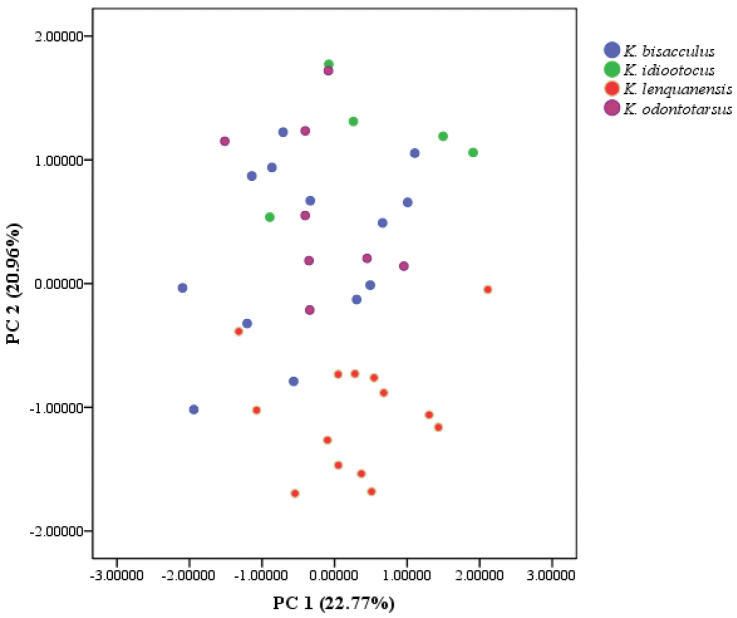
Scatterplot of principal components 1 and 2 of size-adjusted morphometric data for males of *K. lenquanensis* sp. n., *K. idiootocus*, *K. bisacculus*, and *K. odontotarsus*.

**Morphological data.** We retained the first two principal components which accounted for 43.7% of the total variance and had eigenvalues above 1.0. Loadings for PC 1 were all positive except for TD and DNE and were most heavily loaded on HW, IND, and ED (Table 3). No difference was found along the PC 1 axis between the four species. The second principal component (PC 2) accounted for 20.96% of the total variance and loaded heavily and positively on SL and DNE and negatively on FL. Differentiation was found along the PC 2 axis between the new species and *K. idiootocus*, *K. odontotarsus*, and *K. bisacculus* (Fig. 3). The result indicates that the new species differs from *K. idiootocus*, *K. odontotarsus*, and *K. bisacculus* by smaller ratio of SL divided by SVL, smaller ratio of DNE divided by SVL, and higher ratio of FL divided by SVL.

**Table 3.** Factor loadings of the first two principal components of 13 size-adjusted morphometric characteristics of males of *K. lenquanensis* sp. n., *K. idiootocus*, *K. odontotarsus*, and *K. bisacculus*. Absolute values of loading greater than 0.60 in boldface. Abbreviations defined in text.

Character	PC 1	PC 2
Eigenvalue	2.961	2.725
% variation	22.774	20.963
HL	0.376	0.553
HW	**0.715**	0.189
SL	0.369	**0.701**
IND	**0.776**	-0.208
IOD	0.481	-0.443
UEW	0.533	0.416
ED	**0.756**	0.178
TD	-0.19	0.529
FLL	0.437	-0.494
TL	0.362	0.061
FL	0.343	-**0.688**
DNE	-0.068	**0.626**
THL	0.048	-0.232

## Systematics

### Kurixalus lenquanensis sp. n.

Taxon classificationAnimaliaAnuraRhacophoridae

http://zoobank.org/597FB4AE-5B50-4A57-9400-B6EBB36EAC4F

Figs 4 , 5 , 6 , 7

#### Holotype.

**Figure 4.**
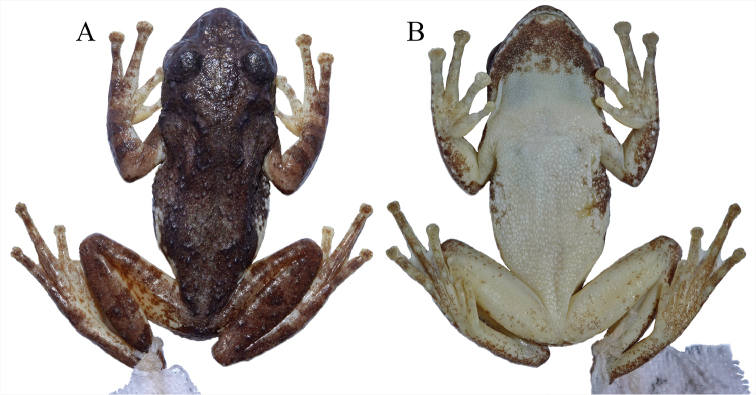
Dorsal **A** and ventral **B** views of the holotype of *Kurixalus lenquanensis* sp. n. in preservative.

**Figure 5.**
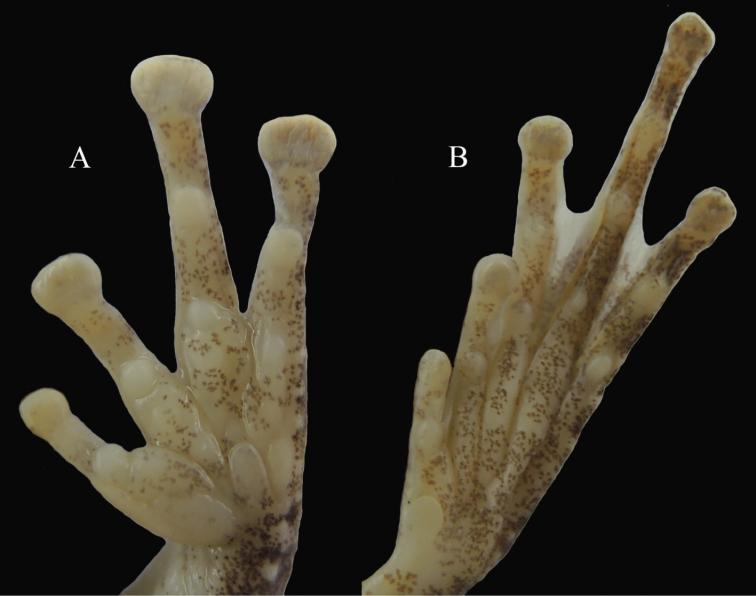
Ventral view of hand **A** and foot **B** of the holotype of *Kurixalus lenquanensis* sp. n. in preservative.

**Figure 6.**
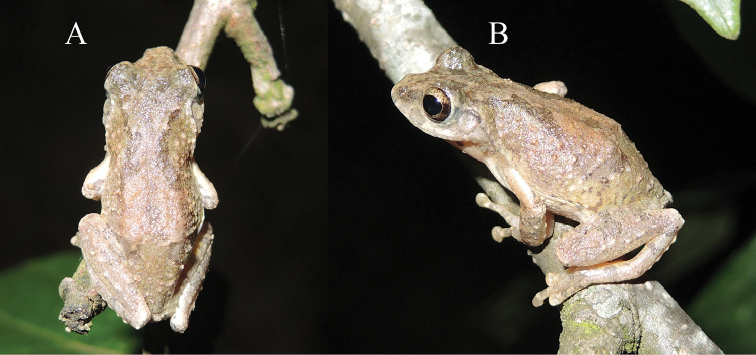
Dorsal **A** and lateral **B** views of the holotype of *Kurixalus lenquanensis* sp. n. in life.

KIZ 170180Y (Figs 4–6), an adult male, collected at 20:35 on 5 May 2016 by Guohua Yu from Lenquan Village (23°12'52"N, 103°22'34"E, 1622 m elevation; Fig. 1), Mengzi City, Yunnan Province, China.

#### Paratype.

Thirteen adult males: KIZ 170175Y–170179Y and KIZ 170181Y–170182Y collected at 20:00–22:45 on 5 May 2016 by Guohua Yu from type locality, KISD 1506203–1506204 collected at 21:00–21:30 on 20 June 2015 by Jishan Wang from the same locality as the holotype, and KIZ 170183Y–170186Y collected at 20:00–22:30 on 6 May 2016 by Guohua Yu from Yangjiatian Village (23°20'5.35"N, 103°9'30.33"E; Fig. 1), Gejiu City, Yunnan Province, China.

#### Type locality.

Lenquan Village, Mengzi City, Yunnan Province, China.

#### Etymology.

The name lenquanensis refers to Lenquan Village, the locality where the new species was found.

#### Diagnosis.

The new tree frog species is assigned to the genus *Kurixalus* based on a combination of the following characters: tips of digits enlarged to discs, bearing circum-marginal grooves; small body size (adult males SVL range of 25.0–28.9 mm; Table 4); finger webbing poorly developed and toe webbing moderately developed; serrated dermal fringes along outer edge of forearm and tarsus; an inverted triangle-shaped dark brown mark between eyes; dorsal brown “) (” saddle-shaped or X-shaped marking maybe present; and coarse dorsal and lateral surfaces with small, and irregular tubercles.

**Table 4.** Measurements (mm) of *K. lenquanensis* sp. n. Abbreviations defined in text.

Vouchers no.	SVL	HL	HW	SL	IND	IOD	UEW	ED	TD	DNE	FLL	TL	FL	THL
KIZ 170175Y	26.7	8.1	9.5	3.6	3.0	3.0	2.6	4.2	1.5	2.0	13.3	12.9	11.7	12.5
KIZ 170176Y	26.1	8.2	9.1	3.6	2.6	2.9	2.6	4.0	1.4	1.9	12.8	12.2	11.6	11.7
KIZ 170177Y	27.4	8.2	9.5	3.7	2.8	2.9	2.5	4.3	1.6	1.6	13.5	12.7	12.2	11.8
KIZ 170178Y	27.1	8.3	9.5	3.9	2.6	2.9	2.5	4.1	1.4	2.0	13.1	13.0	12.0	12.1
KIZ 170179Y	27.3	8.5	9.1	3.5	2.4	2.8	2.5	3.9	1.7	1.9	13.5	12.5	12.2	12.4
KIZ 170180Y	27.2	8.1	9.1	3.7	2.5	2.8	2.3	4.1	1.6	1.7	13.8	12.6	12.5	11.7
KIZ 170181Y	28.9	8.9	9.6	4.0	2.7	2.7	2.6	4.3	1.6	2.0	13.3	12.7	12.4	12.2
KIZ 170182Y	27.1	8.2	9.1	3.7	2.7	2.8	2.5	4.3	1.8	1.6	13.7	11.8	12.7	11.7
KISD 1506203	26.7	8.6	9.3	3.4	2.5	2.9	2.5	4.3	1.6	1.6	13.2	11.9	11.7	11.8
KISD 1506204	27.1	8.9	10.0	3.8	2.8	3.1	2.0	4.0	1.6	2.0	13.1	12.2	11.9	11.9
KIZ 170183Y	26.6	8.4	9.8	3.5	2.5	3.1	2.2	3.9	1.5	1.8	13.2	12.5	12.4	12.2
KIZ 170184Y	26.9	8.7	9.7	3.8	2.8	3.0	2.5	4.3	1.5	1.6	13.1	12.5	12.0	12.2
KIZ 170185Y	27.2	8.8	9.8	3.8	2.7	3.1	2.1	3.9	1.8	1.9	13.4	12.2	11.8	11.2
KIZ 170186Y	25.0	8.7	9.7	3.7	2.6	2.9	2.3	3.9	1.5	1.8	12.4	11.6	11.2	10.8

*Kurixalus lenquanensis* sp. n. can be distinguished from its congeners by a combination of the following characters: smaller body size (mean SVL 27 mm in males); obtusely pointed snout with no prominence on tip; curved canthus rostralis; slight nuptial pad; brown dorsal color; rough dorsum; chin clouded with brown; absence of large dark spots on ventral surface; presence of vomerine teeth; gold brown iris; single internal vocal sac; dermal fringes along outer edge of limbs; rough flanks; and granular throat and chest.

#### Description of holotype.

A small rhacophorid (SVL 27.2 mm); HL 89.4% of HW; snout obtusely pointed, no dermal prominence on tip, projecting slightly beyond margin of lower jaw in ventral view; SL (3.7 mm) shorter than ED (4.1 mm); canthus rostralis blunt and curved; lore region oblique, slightly concave; nostril oval, slightly protuberant, closer to tip of snout than eye; IND (2.5 mm) narrower than IOD (2.8 mm) and slightly wider than UEW (2.3 mm); pineal spot absent; pupil oval, horizontal; tympanum distinct (TD 1.6 mm), rounded, less than half ED; supratympanic fold distinct, curves from posterior edge of eye to insertion of arm; vomerine teeth in two oblique patches touching inner front edges of oval choanae; tongue notched posteriorly; single internal vocal sac.

Limbs slender; relative length of fingers is I < II < IV < III. Tips of all four fingers expanded into discs with circum-marginal and transverse ventral grooves; disc on finger I small, slightly wider than phalanx width; disc width shorter than tympanum width; relative width of discs is I < II < III < IV. Nuptial pad slight; fingers webbed at base, webbing formula is I2–2.5II2–3.5III3–2.5IV following Myers and Duellman (1982). Fringe of skin on edge of all fingers; subarticular tubercles prominent and rounded, formula 1, 1, 2, 2; supranumerary tubercles present; two metacarpal tubercles, outer slightly narrower than inner; row of white warts forming serrated fringe along outer edge of forearm.

Heels overlapping when legs at right angle to body; relative length of toes is I < II < V < III < IV. Tips of toes expanded into discs with circum-marginal and transverse ventral grooves; toe discs smaller than finger discs; disc on toe I same with width as phalanx width; relative size of discs is I < II < III < IV < V. Webbing moderate on all toes, webbing formula is I2–2.5II1.5–3III1.5–3IV2.75–1.5V. Subarticular tubercles prominent and rounded, formula 1, 1, 2, 3, 2; supernumerary tubercles absent; inner metatarsal tubercle distinct, oval; outer metatarsal tubercle absent; series of tubercles forming serrated dermal fringe along outer edge of tarsus and fifth toe.

Numerous small or large tubercles scattered on top of head, upper eyelids, dorsum, and flanks; patch of white tubercles below vent; white conical tubercle on tibiotarsal articulation; throat and chest finely granulated and abdomen coarsely granulated; dorsal surface of limbs tuberculate and ventral surface of thighs finely granulated.

#### Color of holotype in life.

Iris golden brown; dorsal surface grayish brown with dark brown saddle-shaped mark on dorsum, beginning behind eye; dark brown inverted triangle-shaped mark between eyes; lateral head and tympanic region brown with dark brown spot below canthus; broad dark brown bar along canthus rostralis; limbs dorsally brown with clear dark brown barring; rear, anterior, and venter of thigh light yellow with scattered brown spots, more spots on rear; rear of flank faint yellow with brown spots; chest and abdomen white, nearly immaculate; chin clouded with black.

#### Color of holotype in preservative.

In preservative (Fig. 4), dorsal ground color brown, pattern same as in life. Chest and abdomen white; chin clouded with dark brown; flank dirty white with dark brown spots; rear, anterior, and venter of thigh dirty white with scattered brown spots, more so on rear.

#### Variations.

**Figure 7.**
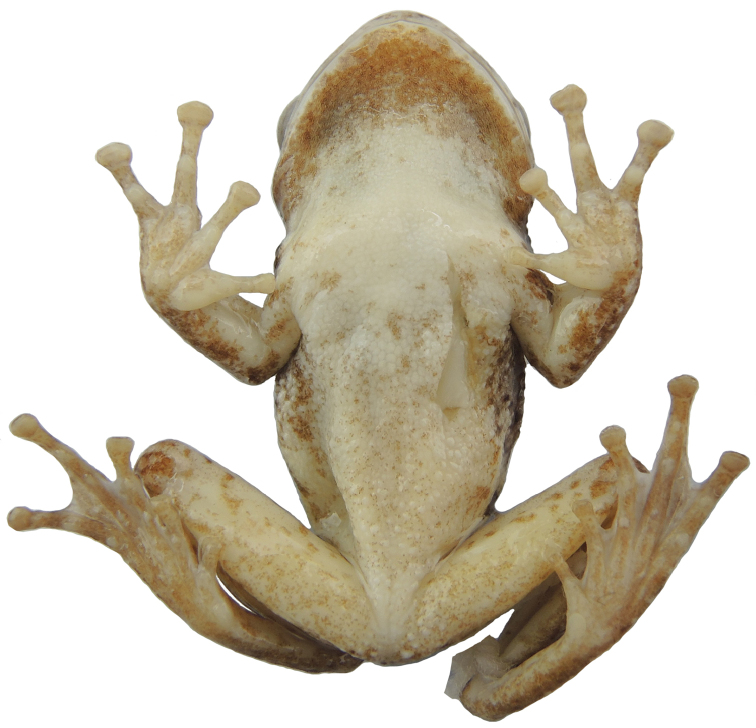
Ventral view of paratype KIZ 170184Y.

Morphometric data are summarized in Table 4. Because the holotype and paratypes of the new species are all male, sexual dimorphism could not be determined. Differing from the nearly immaculate abdominal surfaces of the holotype and paratypes from the type locality, abdominal surfaces of three paratypes (KIZ 170183Y, 170184Y, 170186Y) from Yangjiatian, Gejiu are scattered with fine brown spots (Fig. 7). TL is longer than FL in the holotype and most paratypes, but TL is shorter than FL in paratype KIZ 170182Y (Table 4). Additionally, pattern of dark brown mark on dorsum varies among specimens. The holotype and most paratypes have a saddle-shaped dorsal mark, but the two paratypes KISD 1506203 and KISD 1506204 have an X-shaped dorsal mark and the paratype KIZ 170183Y has no obvious dark brown mark on dorsum.

#### Ecology.

**Figure 8.**
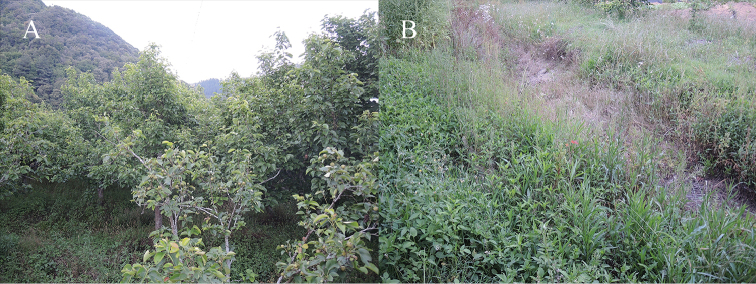
The fruit garden **A** and dry puddle **B** in the fruit garden at type locality of *Kurixalus lenquanensis* sp. n.

At present, the new species is known only from the type locality, Lenquan Village, Mengzi City, Yunnan Province and Yangjiatian Village, Gejiu City, Yunnan Province (Fig. 1). The holotype was found calling on a tree branch approximately 0.5 m above near a dry puddle in a fruit garden in Lenquan Village (Fig. 8). All other specimens were found on vegetation near the dry puddle in Lenquan Village or vegetation near a reservoir in Yangjiatian Village. Males began to call at about 19:30 when sky was getting dark and all specimens were encountered at night (20:00–22:45). Males called loudly, but no females or eggs were found. *Hyla annectans* (Jerdon, 1870), *Kaloula verrucosa* Boulenger, 1904, and *Microhyla heymonsi* Vogt, 1911 were also encountered at the type locality.

#### Comparisons.

**Figure 9.**
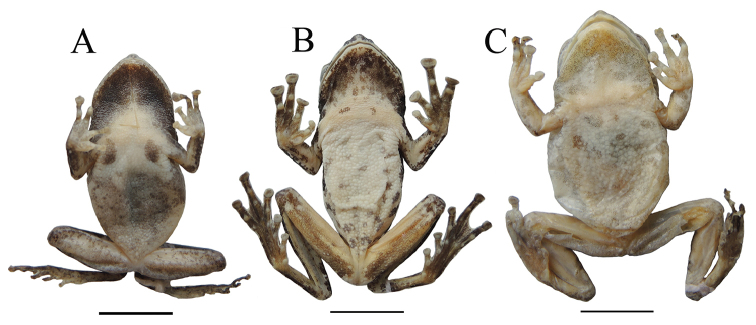
Ventral view of *K. idiootocus* (**A** YGH 140217), *K. odontotarsus* (**B** YGH 090131), and *K. bisacculus* (**C** YGH 090045).

**Figure 10.**
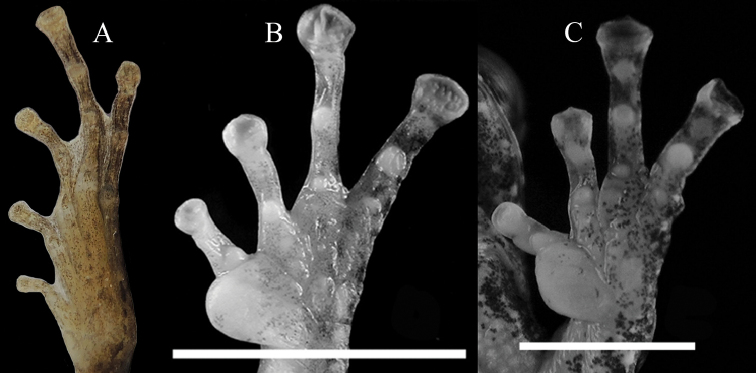
Ventral view of foot of *K. idiootocus* **A** and ventral view of hand of *K. berylliniris* (**B** from Wu et al. 2016) and *K. wangi* (**C** from Wu et al. 2016).

The new species, *Kurixalus lenquanensis* sp. n., is morphologically similar to *K. idiootocus* in that it has a small body size (mean male SVL of 27 mm in new species versus mean male SVL of 27.5 mm in *K. idiootocus*; Table 5). However, the new species can be distinguished from *K. idiootocus* by its obtusely pointed snout with no prominence on tip, absence of a pair of symmetrical large dark patches on chest, single internal vocal sac, and absence of supernumerary plantar tubercles (versus pointed snout with a small prominence on tip, presence of a pair of symmetrical large dark patches on chest, single external vocal sac, and small supernumerary plantar tubercles; Kuramoto and Wang 1987; Figs 9–10). In addition, besides that snout of the new species is shorter than that of *K. idiootocus*, the PCA analysis showed that the new species also differs from *K. idiootocus* by greater ratio of FL divided by SVL (Table 3 and Fig. 3).

**Table 5.** Comparison of measurements (mm) of *K. lenquanensis* sp. n., *K. idiootocus*, *K. odontotarsus*, and *K. bisacculus*. Abbreviations defined in text.

Measurement	*K. lenquanensis* (n = 14)	*K. idiootocus* (n = 5)	*K. odontotarsus* (n = 8)	*K. bisacculus* (n = 13)
SVL	27.0 ± 0.84 (25.0–28.9)	27.5 ± 1.26 (26.1–29)	33.3 ± 0.91 (32.1–34.3)	33.2 ± 0.96 (31.3–34.4)
HL	8.5 ± 0.29 (8.1–8.9)	9.1 ± 0.32 (8.7–9.5)	10.9 ± 0.24 (10.6–11.3)	10.4 ± 0.29 (10.1–10.9)
HW	9.5 ± 0.3 (9.1–10.0)	9.8 ± 0.37 (9.4–10.3)	11.5 ± 0.46 (11.1–12.3)	11.6 ± 0.61 (10.8–12.8)
SL	3.7 ± 0.16 (3.4–4.0)	4.3 ± 0.21 (4.1–4.6)	4.8 ± 0.27 (4.5–5.3)	4.9 ± 0.41 (4.1–5.7)
IND	2.7 ± 0.16 (2.4–3.0)	2.6 ± 0.08 (2.5–2.7)	3.1 ± 0.21 (2.8–3.4)	3.0 ± 0.20 (2.5–3.2)
IOD	2.9 ± 0.13 (2.7–3.1)	2.9 ± 0.21 (2.8–3.3)	3.5 ± 0.17 (3.2–3.6)	3.4 ± 0.21 (3.1–3.6)
UEW	2.4 ± 0.19 (2.0–2.6)	2.8 ± 0.23 (2.5–3.0)	2.9 ± 0.12 (2.8–3.1)	3.0 ± 0.36 (2.6–3.7)
ED	4.1 ± 0.17 (3.9–4.3)	4.4 ± 0.19 (4.1–4.6)	5.1 ± 0.18 (4.8–5.3)	4.8 ± 0.25 (4.4–5.3)
TD	1.6 ± 0.13 (1.4–1.8)	1.7 ± 0.13 (1.6–1.9)	2.3 ± 0.14 (2.1–2.5)	2.2 ± 0.18 (2.0–2.6)
DNE	1.8 ± 0.17 (1.6–2.0)	1.9 ± 0.11 (1.8–2.1)	2.6 ± 0.21 (2.3–2.9)	2.5 ± 0.17 (2.2–2.7)
FLL	13.2 ± 0.36 (12.4–13.8)	12.7 ± 0.43 (12.1–13.3)	16.1 ± 0.57 (14.8–16.5)	15.9 ± 0.37 (15.5–16.8)
TL	12.4 ± 0.41 (11.6–13.0)	12.4 ± 0.47 (11.9–13.1)	15.5 ± 0.64 (14.3–16.1)	15.8 ± 0.6 (14.9–16.9)
FL	12.0 ± 0.41 (11.2–12.7)	10.5 ± 0.36 (9.9–10.9)	13.9 ± 0.97 (12.4–15.2)	13.9 ± 0.76 (13.1–15.9)
THL	11.9 ± 0.46 (10.8–12.5)	11.9 ± 0.56 (11.4–12.8)	15.4 ± 0.75 (14.2–16.2)	15.4 ± 0.93 (13.9–17)

*Kurixalus lenquanensis* sp. n. is distinguished from *K. berylliniris* by gold brown iris, obtusely pointed snout with no prominence on tip, smaller body size, tubercles on upper eyelid, slight nuptial pad, and coarsely granular abdomen (versus emerald to light green iris, pointed snout with a small prominence on tip, larger body size [mean SVL in males = 35 mm], lack of palpebral tubercles, greatly expanded nuptial pad, and smooth abdomen; Wu et al. 2016; Fig. 10).

In addition, the new species can be distinguished from *K. wangi* by a lack of prominence on snout tip, smaller body size, presence of tubercles on dorsum, coarse skin on flanks, and slight nuptial pad (versus pointed snout with small prominence on tip, larger body size [mean SVL in males = 30 mm], absence of tubercles on dorsum, smooth skin on flanks, and greatly expanded nuptial pad; Wu et al. 2016; Fig. 10) and from *K. eiffingeri* by smaller body size, slight nuptial pad, oblique loreal region, and curved canthus rostralis (versus larger body size [mean SVL of 31.1 mm in males], greatly expanded nuptial pad, vertical loreal region, and straight canthus rostralis; Wu et al. 2016) .

*Kurixalus lenquanensis* sp. n. further differs from *Kurixalus appendiculatus* (Günther, 1858) by smaller body size, absence of dermal prominence on snout tip, and tympanum less than half of eye diameter (versus larger body size [male SVL = 30–37 mm], presence of prominence on snout tip, and tympanum half eye diameter; Günther 1858, Inger et al. 1999); from *K. baliogaster* by smaller body size (SVL in males = 25.0–27.4 mm), absence of prominence on obtusely pointed snout tip, absence of large dark spots on ventral surface, tuberculated dorsal and lateral skin, presence of tubercles on eyelids, granular throat, and presence of dermal fringes on limbs (versus larger body size [male SVL = 33.0–33.3 mm], pointed snout with prominence on tip, large dark spots on ventral surface, smooth dorsal and lateral skin, absence of tubercles on eyelids, smooth throat, and absence of dermal fringes on limbs; Inger et al. 1999); and from *Kurixalus banaensis* (Bourret, 1939) by smaller body size, obtusely pointed snout being shorter than eye, presence of vomerine teeth, and tuberculate flanks (versus larger body size [mean SVL in males = 29.7 mm], markedly pointed snout being longer than eye, absence of vomerine teeth, and smooth flanks in *K. banaensis*; Nguyen et al. 2014a, Nguyen et al. 2014b, Bossuyt and Dubois 2001).

The new species differs from *Kurixalus viridescens* Nguyen, Matsui, & Duc, 2014 by tuberculate dorsum, brown dorsal color, dark bands on dorsum and limbs, brownish clouded pattern on chin, and presence of vomerine teeth (versus nearly smooth dorsum, uniformly greenish dorsal color, no dark markings on dorsum and limbs, pinkish cream without marking on chin, and absence of vomerine teeth in *K. viridescens*; Nguyen et al. 2014b); from *Kurixalus ananjevae* (Matsui & Orlov, 2004) by smaller body size, presence of vomerine teeth, presence of dermal fringes on limbs, and finely granular throat surface (versus larger body size [32 mm in one male], absence of vomerine teeth, absence of dermal fringes on limbs, and smooth throat surface; Matsui and Orlov 2004); and from *Kurixalus motokawai* Nguyen, Matsui, & Eto, 2014 by obtusely pointed snout tip, presence of vomerine teeth, and clouded chin with brown (versus pointed snout tip, absence of vomerine teeth, and small dark brown spots scattered on chin; Nguyen et al. 2014a)

Currently, two species of *Kurixalus* (*K. bisacculus* and *K. odontotarsus*) are recognized in Yunnan, China (Yu et al. 2010). The new species can be distinguished from *K. bisacculus* and *K. odontotarsus* by smaller body size, absence of large black spots on belly, and obtusely pointed snout with no prominence on tip (versus larger body size [mean SVL in males at more than 33 mm], presence of large black spots on belly, and markedly pointed snout with a prominence on tip extending beyond lower jaw in *K. bisacculus* and *K. odontotarsus*; Table 5; Fig. 9). Moreover, the PCA analysis revealed that the new species further differs from *K. odontotarsus* and *K. bisacculus* by smaller ratio of SL/SVL, smaller ratio of DNE/SVL, and bigger ratio of FL/SVL (Table 3 and Fig. 3).

Additionally, the new species differs from *K. verrucosus* found in Myanmar by smaller body size, snout shorter than diameter of eye, interorbital distance wider than upper eyelid, tympanum less than half of eye diameter, moderate toe webbing, granular throat and chest, and absence of large brown spots on belly and throat (versus larger body size [mean SVL in males = 29.9 mm], snout as long as diameter of eye, interorbital space as broad as upper eyelid, tympanum half eye diameter, nearly entirely developed toe webbing, smooth throat and chest, and presence of large brown spots on belly and throat; Boulenger 1893); and from *Kurixalus naso* (Annandale, 1912) by smaller body size, obtusely pointed snout with no dermal prominence on tip, moderately developed toe webbing, and absence of large dark spots on chest and belly (versus larger body size [male SVL at more than 30 mm], pointed snout with a dermal prominence on tip, almost completely developed toe webbing, and presence of large dark spots on chest and belly in *K. naso*; Annandale 1912, Mathew and Sen 2008).

## Discussion

Although morphological synapomorphies of the genus *Kurixalus* are still not very clear (Yu et al. 2013, Nguyen et al. 2014b), intuitively *K. lenquanensis* sp. n. can be placed in *Kurixalus* because of its morphological similarity to other members of the genus (e.g. small body size, inner and outer fingers not opposable, poorly developed finger webbing, moderately developed toe webbing, and serrated dermal fringes on forearm and tarsus). Other small rhacophorid species in the genera *Feihyla*, *Gracixalus*, *Chiromantis*, or *Philautus* generally lack serrated fringes on forearm and tarsus and lack vomerine teeth (Fei 1999, Fei et al. 2010). Additionally, inner (first and second) and outer (third and fourth) fingers are opposable in all species of *Feihyla* and *Chiromantis* (Fei 1999, Fei et al. 2010). This assignment is supported by the molecular data, which indicates that *K. lenquanensis* sp. n. is nested in the genus *Kurixalus* with strong support values.

It is very interesting biogeographically that *K. lenquanensis* sp. n. is nested within a clade consisting of Taiwanese *Kurixalus* with strong support, indicating that the ancestor of the new species may have been from Taiwan Island. Another plausible scenario is that the ancestor of this new species may have been widespread in southern China and the descendent species in between Taiwan and Yunnan has become extinct. Although *K. ananjevae* and *K. naso* are not included in the present study, absence of them would have no impact on the phylogenetic position of the new species because *K. ananjevae* likely does not belong to the genus (Yu et al. 2013), and morphologically, *K. naso* is more similar to members of the *K. odontotarsus* species group than to *K. lenquanensis* sp. n. in body size and ventral color pattern.

Reproductive behavior among Taiwanese relatives of the new species varies; *Kurixalus idiootocus* lays pigmented eggs on land near the edge of water or in depressions where rainfall accumulates (Kuramoto and Wang 1987), whereas *K. eiffingeri*, *K. berylliniris*, and *K. wangi* lay eggs inside tree hollows or cut bamboos with water (Fei et al. 2010, Wu et al. 2016). Although no eggs of *K. lenquanensis* sp. n. were found, reproductive behavior of this new species probably is closer to that of *K. idiootocus* than to that of the other three Taiwanese species because 1) it is the sister taxon to *K. idiootocus* and 2) no tree hollows or cut bamboos were found at the type locality.

Species boundaries among members of the genus *Kurixalus* were previously confusing and our earlier work, based on molecular data (Yu et al. 2010), supported that there are three valid members of *Kurixalus* in mainland China. Therefore, with the new species described here, there are currently four *Kurixalus* species in mainland China: *K. bisacculus*, *K. lenquanensis* sp. n., *K. odontotarsus*, and *K. verrucosus*. However, considering the obvious geographical discontinuity in distribution between *K. lenquanensis* sp. n. and its congeneric relatives from Taiwan Island, additional undiscovered species of *Kurixalus* may exist in south China.

### Key to the new species and its congeners

**Table:** 

1	Limbs with no serrated dermal fringes	**2**
–	Limbs with serrated dermal fringes	**3**
2	Dorsum smooth; many dark spots scattered on ventral surface	***K. baliogaster***
–	Dorsum with small tubercles, no dark spots on ventral surface	***K. ananjevae***
3	Dorsal color uniformly greenish	***K. viridescens***
–	Dorsal color not uniformly greenish, generally brownish mixed with darkmarking	**4**
4	Iris emerald to light green	***K. berylliniris***
–	Iris golden	**5**
5	Nuptial pad greatly expanded	**6**
–	Nuptial pad slight	**7**
6	Tubercles on lateral margin of finger IV connected with dermal fringe; venter whitish with very little pigmentation; loreal region oblique; canthus rostralis curved	***K. wangi***
–	Tubercles on lateral margin of finger IV separated from each other; venter with numerous fine brownish dots, especially in the gular region; loreal region vertical; canthus rostralis straight	***K. eiffingeri***
7	Vomerine teeth absent	**8**
–	Vomerine teeth present	**9**
8	Snout tip less markedly pointed; lateral fringes on limbs and infra-cloacal tubercles less developed; lateral sides areolate	***K. motokawai***
–	Snout tip markedly pointed; lateral fringes on limbs and infra-cloacal tubercles developed; flanks smooth	***K. banaensis***
9	Smaller body size (adult male SVL less than 30 mm)	**10**
–	Bigger body size (generally adult male SVL greater than 30 mm)	**11**
10	Snout obtusely pointed with no prominence on tip; absence of a pair of symmetrical large dark patches on chest; single internal vocal sac	***K. lenquanensis* sp. n.**
–	Snout pointed with a small prominence on tip; a pair of symmetrical large dark patches present on chest; single external vocal sac	***K. idiootocus***
11	Snout rounded or somewhat pointed; chin and breast smooth	***K. verrucosus***
–	Snout obviously pointed; chin and breast granular	**12**
12	Dorso-lateral fold fairly distinct	***K. naso***
–	No obvious dorso-lateral fold	**13**
13	Venter uniformly cream to tan with no black spots	***K. appendiculatus***
–	Black spots present on ventral surface	**14**
14	Single internal vocal sac; omosternum unforked	***K. odontotarsus***
–	Paired lateral vocal sacs or single internal vocal sac and omosternum forked	***K. bisacculus***

### Comparative material examined

*Kurixalus idiootocus*: YGH 140215, 140217–140220, Xinbei, Taiwan.

*Kurixalus odontotarsus*: YGH 090130–090137, Caiyanghe, Puer, Yunnan.

*Kurixalus bisacculus*: YGH 080166, 080168–080170, 140013, Pingbian, Yunnan; YGH 090045, 140020, Wenshan, Yunnan; YGH 090081, Libo, Guizhou; YGH 090202, Longmeng, Guangdong; YGH 090268, 090270, Nanning, Guangxi; THNHM 10051, 10052, Nan, Thailand.

*Kurixalus verrucosus*: CAS 225128, 231489, 231491, 224563, Kachin, Myanmar.

## Supplementary Material

XML Treatment for Kurixalus lenquanensis
